# Effect of Lipid Oxidation Products on Histamine Formation in Fermented Sausages

**DOI:** 10.3390/foods14234166

**Published:** 2025-12-04

**Authors:** Yuxiang Xu, Huaihui Zhang, Hongzhan Wang, Wei Zhang, Yilun Wang

**Affiliations:** Laboratory of Food Science and Engineering, College of Life Sciences and Agri-Forestry, Southwest University of Science and Technology, No. 59, Middle Qinglong Avenue, Mianyang 621010, China; 18081910253@163.com (Y.X.);

**Keywords:** fermented sausage, histamine, lipid oxidation, correlation analysis

## Abstract

This study aimed to investigate the influence of lipid oxidation intensity on histamine accumulation in fermented sausages and to identify specific lipid oxidation products potentially responsible for promoting histamine formation. Pork backfat was subjected to controlled oxidation at different temperatures to obtain varying degrees of oxidation and subsequently used in fermented sausage production. Histamine content was determined, and treatment groups exhibiting significant differences (50 °C, 60 °C, and 70 °C) were selected for further non-targeted metabolomic analysis. Multivariate statistical approaches, including PCA and OPLS-DA, were employed to screen and identify differential lipid oxidation products. The results revealed a significant positive correlation between the extent of lipid oxidation and histamine content (*p* < 0.05). Within the selected temperature range, the mean histamine contents were 22.97, 19.05, and 17.45 mg/kg for the 70 °C, 60 °C, and 50 °C treatments, respectively. A total of 33 lipid oxidation products were identified during ripening, predominantly aldehydes, ketones, alcohols, esters, acids, and alkanes. Pearson correlation analysis showed that four compounds—decanal, nonanal, hexanal, and 1-octen-3-ol—were strongly and positively correlated with histamine content (*p* < 0.01). These compounds were highlighted as key lipid-derived contributors potentially associated with elevated histamine levels. This study provides initial evidence that specific lipid oxidation products are closely linked to histamine accumulation in a real fermented sausage matrix. The findings offer a theoretical basis for reducing biogenic amine formation by controlling lipid oxidation during processing, which could have important practical implications for improving the safety of fermented meat products.

## 1. Introduction

Fermented sausages are traditional fermented meat products highly valued by consumers for their attractive color, distinctive flavor, rich texture, and high nutritional value [1]. However, biogenic amines, which pose potential health risks to humans, can be formed during processing and storage, with histamine recognized as the most toxic among them [2]. Lipid oxidation is a major factor influencing the quality of meat products. Moderate lipid oxidation contributes positively to the development of desirable color and characteristic flavor [3], whereas excessive oxidation not only compromises sensory and nutritional quality but may also adversely affect consumer health.

Research suggests that in fermented foods, biogenic amines are formed not only through microbial enzymatic decarboxylation of amino acids catalyzed by decarboxylases and produced by amine-forming bacteria, but also via a non-enzymatic chemical pathway in which free amino acids undergo decarboxylation in the presence of certain lipid oxidation products [4,5,6]. Zamora et al. [4] demonstrated that phenylalanine undergoes decarboxylation in the presence of common lipid oxidation products (e.g., methyl 13-hydroperoxyoctadeca-9,11-dienoate and 2,4-decadienal) to yield β-phenylethylamine. Decarboxylation proceeds as follows: initially, the amino group of phenylalanine reacts with the carbonyl group of the lipid oxidation product to form an unstable 5-oxazolinone intermediate, which readily eliminates a molecule of carbon dioxide to generate a relatively stable methylenimine ylide. Subsequently, the methylenimine ylide reacts with water to produce a novel conjugated imine. Finally, the conjugated imine undergoes a series of transformations to afford β-phenylethylamine. Hidalgo et al. [5] investigated the decarboxylation of histidine to histamine in the presence of lipid oxidation products and found that, among the various products examined, 2,4-alkadienals were the most reactive in promoting this decarboxylation, exhibiting the lowest activation energy required for histidine’s conversion to histamine. Most previous studies have been conducted in model systems or pure chemical environments. However, fermented meat products represent highly complex food matrices in which interactions among proteins, lipids, microorganisms, and endogenous enzymes may significantly alter reaction pathways. To elucidate which classes or specific lipid oxidation products are associated with enhanced histamine accumulation in real fermented sausages, pork backfat was subjected to controlled thermal oxidation at different temperatures to obtain varying degrees of oxidation. The resulting backfat was subsequently incorporated into fermented sausage formulations. Histamine content was quantified throughout ripening, and treatments exhibiting significant differences (50 °C, 60 °C, and 70 °C) were selected for non-targeted lipidomic analysis. Multivariate statistical approaches, including PCA and OPLS-DA, were then applied to identify differential lipid oxidation products and establish their correlations with histamine content. This study provides the first comprehensive investigation, within an actual fermented sausage system, of the relationship between the extent of lipid oxidation and histamine accumulation, while pinpointing specific lipid-derived compounds most strongly associated with elevated histamine concentrations. These findings offer a more accurate assessment of the potential contribution of lipid oxidation to biogenic amine risk and establish a critical theoretical basis for developing targeted strategies to control lipid oxidation, thereby reducing biogenic amine formation and enhancing the safety of fermented meat products.

## 2. Materials and Methods

### 2.1. Preparation of Pork Backfat with Different Degrees of Oxidation

Fresh pork backfat was purchased from a local wholesale market in Mianyang, China. Visible connective tissue and lean meat were removed, and the backfat was rinsed with deionized water. It was then blanched in boiling water for 10 s to inactivate lipases, followed by immediate cooling in ice water. The outer surface layer (approximately 2 cm thick) was discarded to minimize microbial contamination. On a disinfected workbench, the backfat was sliced into uniform pieces (3 cm × 2 cm × 0.2 cm) using a sterilized knife. The slices were thoroughly mixed, and 200 g aliquots were placed into oxygen-permeable sterile homogenization bags (Anke Biological Products Co., Ltd., Shanghai, China). Following a modified method from Liu et al. [7], the bags were randomly assigned to seven treatment groups and incubated at −80 °C, −20 °C, 40 °C, 50 °C, 60 °C, 70 °C, or 80 °C for 6 h in temperature-controlled ovens (S881, Suzhou Huajie Oven Manufacturing Co., Ltd., Suzhou, China) or freezers (DW-86L, Haier, Qingdao, China). After treatment, the backfat was immediately minced under aseptic conditions using a sterile meat grinder (S18-LA170, Joyoung Co., Ltd., Shanghai, China) equipped with a 4 mm plate to ensure homogeneity. Subsamples were collected for subsequent determination of peroxide value (POV) and thiobarbituric acid reactive substances (TBARSs), as well as for fermented sausage production.

### 2.2. Determination of Oxidation Index

#### 2.2.1. POV

The peroxide value was determined according to the International Standard ISO 3960:2017 [8] with minor modifications. Briefly, a 50 g of homogenized backfat was extracted with 150 mL of petroleum ether (boiling range 30–60 °C) for 12 h in the dark at room temperature (20 °C). The filtrate was then heated in a water bath at below 40 °C (XMTD-7000, Beijing Yongguangming Medical Instrument Co., Ltd., Beijing, China) until it reached a constant weight. An accurately measured 2 g of the filtrate was transferred into a mixture of 30 mL chloroform–acetic acid and 1 mL saturated potassium iodide solution, and reacted in the dark for 3 min. Subsequently, 1 mL of starch indicator was added, and the solution was titrated with 2 mmol/L sodium thiosulfate standard solution until the blue color disappeared.

#### 2.2.2. TBARS

TBARS values were determined following the method of Li et al. [9] with slight modifications. Briefly, 5.00 g of homogenized sample was mixed with 3 mL of 1% (*w*/*v*) 2-thiobarbituric acid (TBA) solution and 17 mL of 2.5% (*v*/*v*) trichloroacetic acid–hydrochloric acid (TCA–HCl) solution. The mixture was heated in a boiling water bath (100 °C) for 30 min, then rapidly cooled to room temperature under running tap water. The supernatant was mixed with an equal volume of chloroform, centrifuged at 4000 rpm (5804R, Eppendorf Co., Ltd., Hamburg, Germany) for 10 min, and the supernatant was collected. Absorbance at 532 nm (UV-1800PC, Shanghai Lengguang Technology Co., Ltd., Shanghai, China) (*A*_532 nm_) was measured, and the TBARS value was calculated using the following equation:

$$\mathrm{TBARS}\;(\mathrm{mg}/\mathrm{kg})\;=\frac{A_{532}}{W_{S}}\;\times \;9.48$$

where $A_{532}$ is the absorbance of the test solution, $W_{s}$ is the sample mass (mg/g), and 9.48 is a constant derived from the TBARS dilution factor and the molar extinction coefficient.

### 2.3. Production of Fermented Sausage Production

The formulation of Sichuan sausage (per 100 kg of total meat batter) consisted of 70 kg lean pork, 30 kg pork backfat, 2.5 kg salt, 1.0 kg chili powder, 1.0 kg white sugar, 1.0 kg Chinese Baijiu (50% vol), 0.4 kg Sichuan pepper powder, 0.15 kg monosodium glutamate, and 0.05 kg mixed spices (star anise, clove, and cinnamon powder). All raw materials and ingredients were purchased from Yonghui Supermarket (Mianyang, China).Lean pork and the pre-oxidized pork backfat obtained from the −20 °C, 50 °C, 60 °C, 70 °C, and 80 °C treatments were minced separately through a 6 mm plate. The minced lean pork was thoroughly mixed with the corresponding oxidized backfat and all seasonings according to the above recipe to ensure homogeneity. The resulting meat batter was held at 4 °C for 4 h to allow flavor penetration and salt diffusion. Subsequently, the mixture was stuffed into natural pork casings (diameter 28–30 mm) using a hydraulic stuffer (SGC-3, Guangdong Henglian Food Machinery Co., Ltd., Foshan, China). The filled sausages were then transferred to a fermentation chamber and fermented at 20 °C and 75% relative humidity (RH) for 48 h. After fermentation, the sausages were moved to a ripening room and matured at 13 °C and 60% RH for 28 days. Sampling was performed at 0, 8, 16, 20, 24, and 30 days of the entire fermentation and ripening period, and the samples were immediately vacuum-packed and stored at −80 °C until analysis.

### 2.4. Determination of Histamine Content and Selection of Groups with Significant Differences

Histamine content was determined by high-performance liquid chromatography (HPLC) with fluorescence detection following the dansyl chloride derivatization method of Sun et al. [10] with minor modifications. Briefly, 5.00 g of homogenized sausage sample was extracted twice with 20 mL of 0.4 mol/L perchloric acid. Each extraction was followed by centrifugation at 8000× *g* and 4 °C for 10 min. The combined supernatants were adjusted to 50 mL with 0.4 mol/L perchloric acid. A 1.00 mL aliquot of the extract was sequentially mixed with 200 μL of 2 mol/L NaOH, 300 μL of saturated NaHCO_3_ solution, and 2 mL of dansyl chloride solution (10 mg/mL in acetonitrile). After thorough vortexing, derivatization was performed at 40 °C in the dark for 45 min. The reaction was terminated by adding 100 μL of 25% (*v*/*v*) ammonia solution. The final volume was adjusted to 5 mL with acetonitrile, and the solution was filtered through a 0.22 μm PTFE syringe filter (Tianjin Jinteng Experiment Equipment Co., Ltd., Tianjin, China). The derivatized samples and histamine standards (prepared identically) were analyzed using an Agilent 7820A HPLC system equipped with a fluorescence detector (Agilent Technologies, Santa Clara, CA, USA).

Chromatographic Conditions: C_18_ column (4.6 × 250, 5 μm) (Agilent Technologies, Palo Alto, CA, USA) was used under the following conditions: flow rate 0.8 mL/min, UV detector wavelength 254 nm, injection volume 10 μL, column temperature 30 °C. The mobile phases were: A = ultra-pure water and B = chromatographic-grade acetonitrile. The gradient elution program was as follows: 0 min, 35%A + 65%B; 5 min, 30%A + 70%B; 20 min, 0%A + 100%B; 25 min, 35%A + 65%B, held for 30 min.

### 2.5. Determination of Lipid Oxidation Product Categories

Lipid oxidation products were determined following the method of Olivares et al. [11] with minor modifications. Briefly, 10.00 g portion of the meat sample was thoroughly mixed with 0.75 g of butylated hydroxytoluene (BHT) and 1 g of sodium chloride (NaCl), then sealed in a headspace extraction vial. The sealed vial was equilibrated in a water bath at 80 °C for 30 min, after which a solid-phase microextraction (SPME) fiber was inserted into the vial and allowed to extract volatile compounds for 30 min. The fiber was then removed and analyzed by GC–MS (5975C, Agilent Technologies, Palo Alto, CA, USA).

Chromatographic conditions: The GC was equipped with an Agilent HP-5MS capillary column (30 m × 250 μm × 0.5 μm). Helium was used as the carrier gas at a flow rate of 1.2 mL/min. The oven temperature program was as follows: hold at 45 °C for 4 min; increase at 1 °C/min to 60 °C and hold for 1 min; increase at 4 °C/min to 150 °C and hold for 3 min; then increase at 10 °C/min to 260 °C. The injector temperature was set at 250 °C. Ionization mode: electron impact (EI); ion source temperature: 230 °C; quadrupole temperature: 150 °C; acquisition mode: full scan. The comparison of the retention times of lipid oxidation products with the NIST spectral library, and their identification based on retention indices (RIs) > 890.

### 2.6. Statistical Analysis

Data are expressed as mean ± standard deviation from three independent replicates. Statistical analyses were performed using SPSS 23.0 (IBM Corp., Armonk, NY, USA). Histamine content was analyzed by one-way analysis of variance (ANOVA) followed by Tukey’s honestly significant difference (HSD) test; groups exhibiting significant differences were selected for subsequent metabolomic analysis. For other multiple comparisons, Duncan’s multiple range test was applied, with statistical significance established at *p* < 0.05 and high significance at *p* < 0.01.

Multivariate statistical analyses, including principal component analysis (PCA) and orthogonal partial least squares discriminant analysis (OPLS-DA), were conducted using SIMCA 14.1 software (Sartorius Stedim Data Analytics AB, Umea, Sweden). Differential lipid oxidation products were screened based on the following criteria: Variable Importance in Projection (VIP) ≥ 1, *p* < 0.05, and Fold Change (FC) ≥ 1.5. To evaluate the relationship between these differential products and histamine content, Pearson’s correlation analysis was performed, and heatmaps were generated using TBtools (version 0.665). All graphs were plotted using Origin 2023 (OriginLab Corp., Northampton, MA, USA).

## 3. Results and Discussion

### 3.1. POV and TBARS

**Figure 1:**
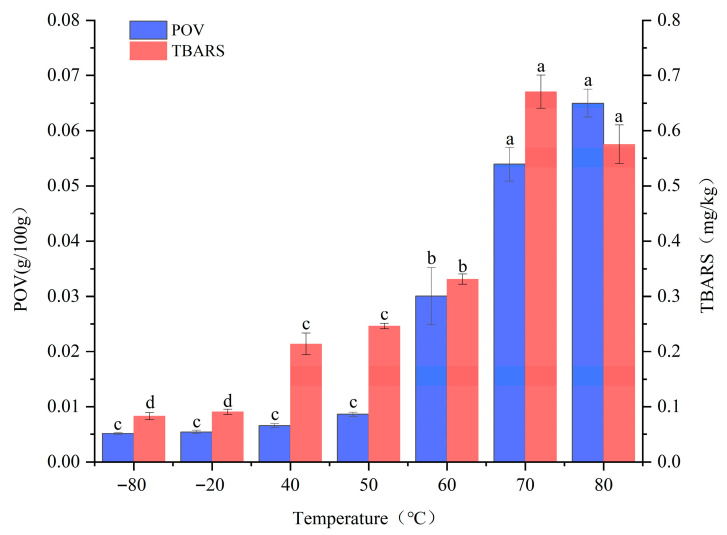
Effect of different temperatures on POV and TBARS. Note: Different lowercase letters within the same indicator represent significant differences (*p* < 0.05) at different temperatures.

POV and TBARS are widely used as primary indicators for assessing the extent of lipid oxidation. POV measures the concentration of primary oxidation products (mainly hydroperoxides) [12], whereas TBARS quantify secondary oxidation products, predominantly aldehydes such as malondialdehyde [13]. As shown in Figure 1, both POV and TBARS values remained low at −80 °C and −20 °C. Within the 50–70 °C range, POV increased significantly (*p* < 0.05), followed by a more gradual rise from 70 °C to 80 °C. TBARS exhibited a marked increase (*p* < 0.05) between 40 °C and 60 °C, reached a maximum at 70 °C, and then declined slightly from 70 °C to 80 °C.

At low temperatures, minimal changes in POV and TBARS may be attributed to the near-complete inactivation of pro-oxidative enzymes such as lipoxygenase below −20 °C, along with the strong inhibition of lipid diffusion and radical chain reactions under ultra-low temperatures [14]. In the 40–60 °C range, radical chain reactions accelerate, leading to the generation of abundant primary oxidation products and a corresponding increase in POV. Meanwhile, hydrogen peroxides begin decomposing into aldehydes (e.g., malondialdehyde), resulting in an increase in TBARS levels [3]. At 80 °C, TBARS values showed a decreasing trend, consistent with the findings of Del Pulgar et al. [15], likely due to the almost complete inactivation of pro-oxidative enzymes such as lipoxygenase and the rapid degradation of secondary oxidation products. In summary, high-temperature treatment markedly accelerates the oxidation process in pork back-fat.

### 3.2. Histamine Content

**Figure 2:**
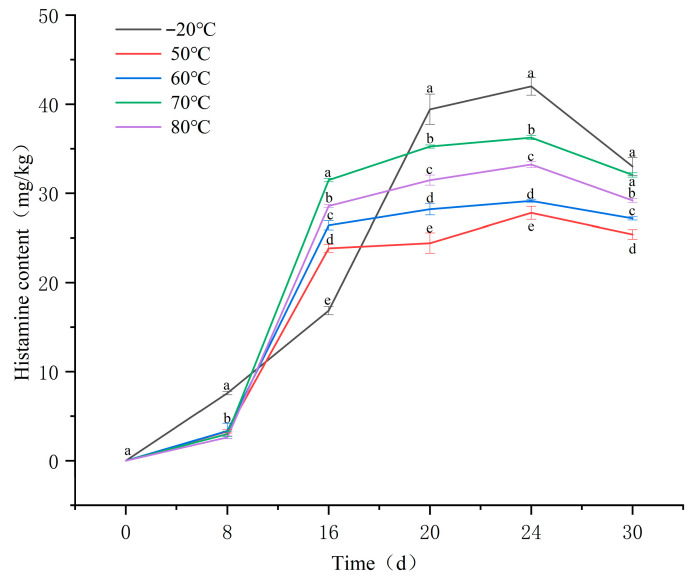
Histamine content in fermented sausages. Note: Different lowercase letters within the same indicator represent significant differences (*p* < 0.05) at different time points.

Changes in histamine content during the ripening of fermented sausages are presented in Figure 2. In the groups subjected to 50 °C, 60 °C, 70 °C, and 80 °C, histamine content remained low during the early ripening stage (0–8 days), after which it increased rapidly from day 8 onward and reached maximum levels around day 20. Notably, the accumulation in the −20 °C group was relatively slow during the early stage of ripening but exhibited a marked increase in the mid-to-late phase, ultimately resulting in the highest final histamine content among all groups. This phenomenon can be primarily attributed to two reasons: (1) extensive proteolysis mediated by endogenous proteases (released or activated during freeze–thaw-induced tissue damage), which generated a substantial pool of free histidine as a precursor; and (2) the amine-producing microorganisms were temporarily inactivated at −20 °C but regained activity during the later stages of maturation, thereby promoting histamine formation during sausage fermentation [7,16,17].

By 20 d of ripening, histamine content in the 70 °C and 80 °C groups was significantly higher than that in all other temperature groups, except for the −20 °C group (*p* < 0.05). This pattern highlights a positive association between histamine accumulation and the extent of lipid oxidation, which aligns with the observations reported by Zhou et al. [18]. Similarly, Liu et al. [7] demonstrated that increases in lipid oxidation indicators (POV and TBARS values) were accompanied by significant elevations in the levels of several biogenic amines, including histamine (ranging from 25.82 to 34.97 mg/kg).

During the ripening period, the final histamine content decreased in the following order: −20 °C > 70 °C > 80 °C > 60 °C > 50 °C, with statistically significant differences observed among the treatment groups (*p* < 0.05). Within the temperature range of 50–70 °C, histamine content in fermented sausages showed a significant positive correlation with the degree of lipid oxidation (*p* < 0.05). This finding indicates that certain lipid oxidation products generated under these conditions may contribute to histamine accumulation. To identify the specific lipid oxidation products associated with elevated histamine levels, the three temperature treatments exhibiting marked differences in histamine content (50 °C, 60 °C, and 70 °C) were selected for further metabolomic analysis of lipid oxidation products.

### 3.3. Analysis of Lipid Oxidation Product Differences in Fermented Sausages

#### 3.3.1. Lipid Oxidation Products

To further investigate which specific lipid oxidation products or categories promote histamine formation in sausages at the three temperature groups (50 °C, 60 °C, and 70 °C) where histamine content showed significant differences, gas chromatography–mass spectrometry (GC-MS) was used to identify lipid oxidation products. Database searches and spectrum matching were performed to determine the types of lipid oxidation products, and their relative abundances were calculated using a normalization method based on peak areas. Lipid oxidation products from fermentation days 16, 20, and 24, when histamine content differed significantly (*p* < 0.05), were selected for analysis. The types and relative abundances of lipid oxidation products at different temperatures are presented in Table 1. Lipid oxidation products in fermented sausages were mainly composed of aldehydes, ketones, esters, alcohols, acids, and alkanes. As shown in Table 1, aldehydes were dominated by nonanal, decanal, and hexanal; ketones by acetylacetone, cedrene, and heptadecanone; esters were dominated by ethyl hexanoate, ethyl decanoate, and diethyl phthalate; alcohols were dominated by 1-octanol and 1-hexanol; acids were primarily dominated by acetic acid; and alkanes were mainly dominated by tetradecane and hexadecane. The minor lipid oxidation products in fermented sausages are shown in Table S1 of the Supplementary Materials.

With increasing temperature, the overall aldehyde content exhibited an upward trend, consistent with the findings of Xia et al. [19]. Compared to storage at 50 °C, the total aldehyde content at 70 °C was significantly higher, exhibiting 3.52-fold (95% CI: 2.90–4.11), 2.36-fold (95% CI: 2.22–2.50), and 3.91-fold (95% CI: 3.79–4.03) increases by 16 d, 20 d, and 24 d, respectively. Two-way ANOVA revealed a very large effect of temperature on total aldehyde formation (η^2^ = 0.913), indicating that temperature accounted for 91.3% of the variance. Such pronounced elevation is primarily attributable to the accelerated autoxidation of lipids via free-radical chain reactions at elevated temperatures, coupled with thermal disruption of adipose cell membranes, which facilitates the release of polyunsaturated fatty acids and other precursors that subsequently undergo oxidative cleavage to form aldehydes [19,20,21]. Similarly, several ketones (e.g., acetylacetone and β-cedrene) and esters (e.g., ethyl hexanoate and ethyl decanoate) exhibited significant increases (*p* < 0.05) with rising temperature, although the magnitude was less dramatic than that observed for aldehydes. At 70 °C, the total ketone and ester contents increased by 1.01- to 2.94-fold relative to 50 °C. In particular, the total ester content showed an exceptionally large temperature effect (η^2^ = 0.99), explaining nearly all observed variance. Notably, some compounds that were undetectable at lower temperatures, such as 1-octanol and 1-octen-3-ol, emerged and increased significantly (*p* < 0.05) in concentration at 70 °C. Overall, elevated temperatures primarily promoted the accumulation of lipid oxidation products, particularly aldehydes, with the most pronounced effects observed at 70 °C.

#### 3.3.2. Principal Component Analysis (PCA)

**Figure 3:**
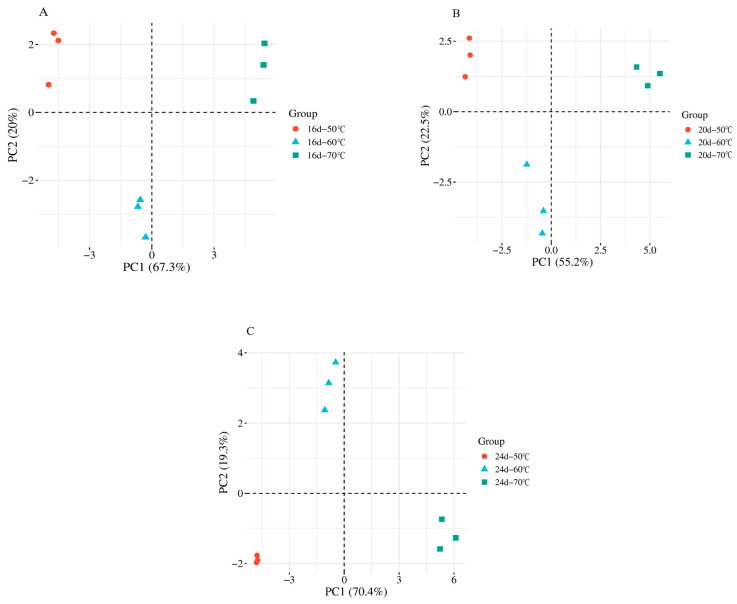
The PCA score plots of lipid oxidation products in fermented sausages under different temperatures. (**A**–**C**) represent the samples taken on 16 d, 20 d, and 24 d, respectively.

PCA was performed on the lipid oxidation products from the aforementioned temperature groups to simplify complex datasets while retaining the maximum amount of original information, thereby reflecting the inherent structure of the data [22]. The PCA score plots visually illustrate the differences among samples, as shown in Figure 3, where A–C represent samples collected on 16 d, 20 d, and 24 d, respectively.

At 16 d, the first principal component (PC1) and second principal component (PC2) accounted for 67.3% and 20.0% of the total variance, respectively. At 20 d, PC1 and PC2 contributed 55.2% and 22.5%, respectively, while at 24 d, PC1 and PC2 contributed 70.4% and 19.3%. For all three fermentation stages, the cumulative contribution rates (PC1 + PC2) exceeded 70%, indicating that the PCA model effectively captured most of the variance in the dataset. PCA visualizes differences among components based on variations in signal intensity, and when the cumulative contribution rate exceeds 60%, the PCA model is generally considered valid [23].

Furthermore, the dense clustering of samples with the same color and the clear separation of different colors into distinct quadrants indicate high reproducibility and significant differences in lipid oxidation products among fermented sausages treated at different temperatures. These results support the statistical findings presented in Table 1, providing a solid foundation for subsequent analyses.

#### 3.3.3. Orthogonal Partial Least Squares-Discriminant Analysis (OPLS-DA)

OPLS-DA was performed on the lipid oxidation products from different temperature groups at three sampling time points. An OPLS-DA score plot was constructed, where each point represents a sample, and samples within the same group are marked with the same color.

**Figure 4:**
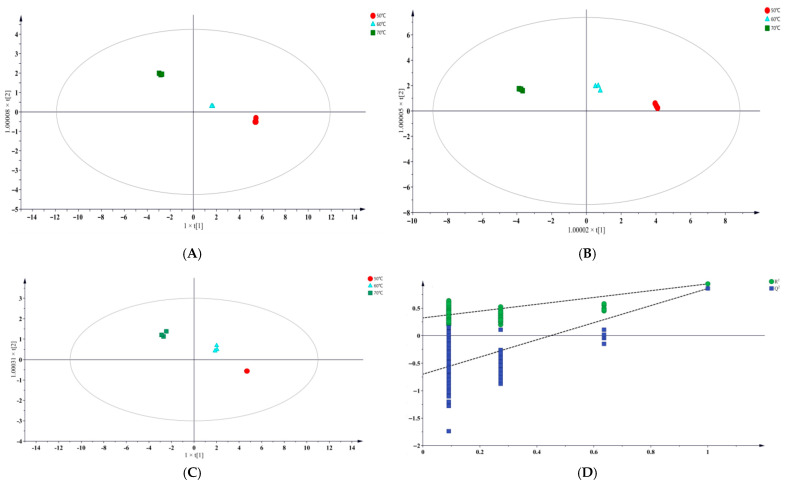
The OPLS-DA score plots of the sample groups. (**A**–**C**) represent the samples taken on 16 d, 20 d, and 24 d, respectively, and (**D**) represents the permutation test of the OPLS-DA model.

At 16 d, the three temperature groups showed clear separation in the OPLS-DA score plot, with partial overlap among the groups (Figure 4A). The 50 °C and 60 °C groups were relatively close to each other, suggesting that the levels of lipid oxidation products were similar under these two temperature conditions [24]. In contrast, the 70 °C group deviated markedly from the 50 °C group, occupying a distinct quadrant, indicating that higher temperature treatment significantly altered the composition of lipid oxidation products.

At 20 d (Figure 4B), the 70 °C group was clearly separated from the other temperature groups, although partial overlap remained. By 24 d (Figure 4C), the OPLS-DA score plot showed a clear and statistically significant separation (*p* < 0.05) among temperature groups, while the difference between the 50 °C and 60° C groups decreased.

Parallel samples were tightly clustered in all plots, demonstrating good experimental reproducibility and model stability. A subsequent permutation test (200 iterations) (Figure 4D) yielded R^2^ = 0.323 and Q^2^ = −0.698, confirming that the OPLS-DA model was valid and free from overfitting [25]. Therefore, this model was suitable for screening differential lipid oxidation products across temperature treatments.

#### 3.3.4. Screening of Differential Lipid Oxidation Products

PCA and OPLS-DA were applied to analyze lipid oxidation products in fermented sausages prepared under different temperature treatments. Differential lipid oxidation products were selected based on three criteria: Variable Importance in Projection (VIP) ≥ 1, *p* < 0.05, and Fold Change (FC) ≥ 1.5. A higher VIP value indicates a more significant difference in lipid oxidation products among temperature treatments. [26].

**Figure 5:**
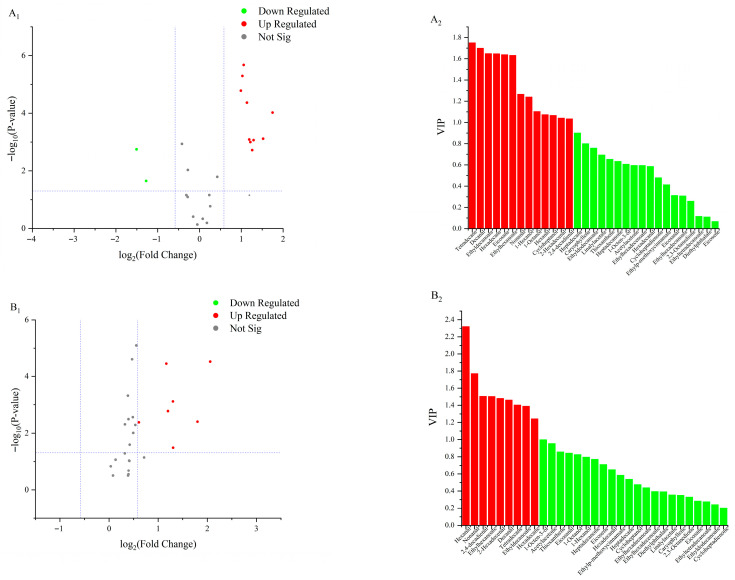

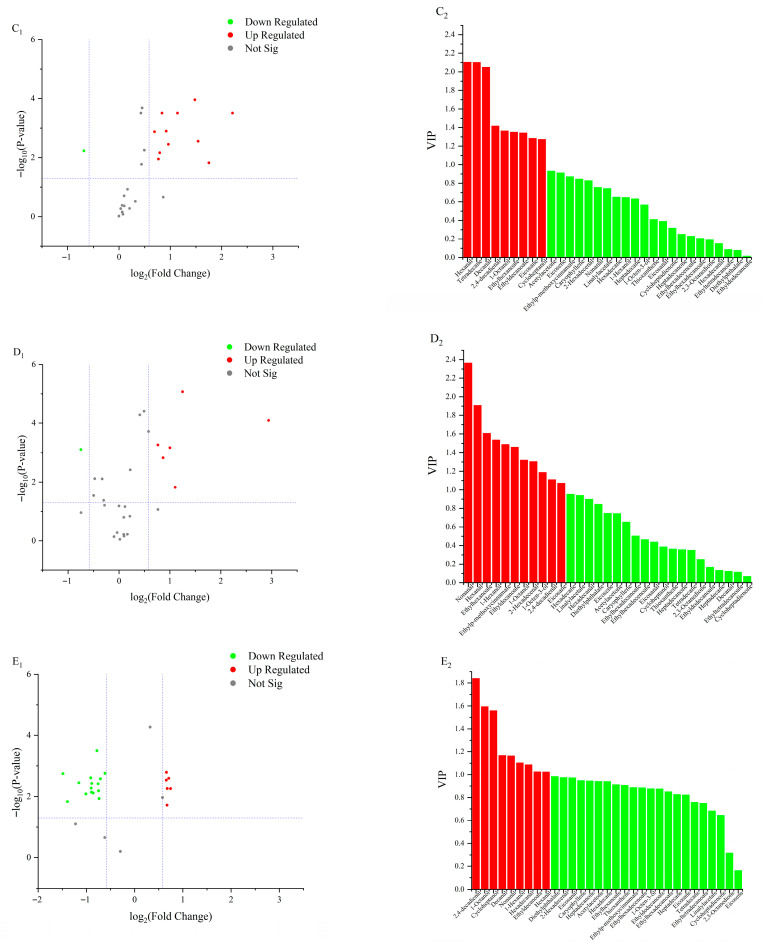

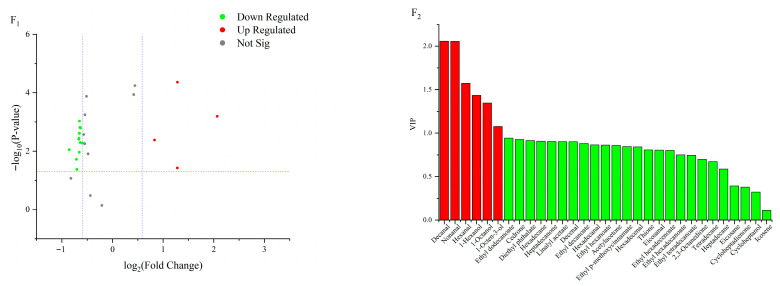
Volcano plots and VIP value plots of lipid oxidation products. (**A1**,**A2**) represents the comparison between samples at 50 °C and 60 °C on 16 d; (**B1**,**B2**) represents the comparison between samples at 60 °C and 70 °C on 16 d; (**C1**,**C2**) represents the comparison between samples at 50 °C and 60 °C on 20 d; (**D1**,**D2**) represents the comparison between samples at 60 °C and 70 °C on 20 d; (**E1**,**E2**) represents the comparison between samples at 50 °C and 60 °C on 24 d; (**F1**,**F2**) represents the comparison between samples at 60 °C and 70 °C on 24 d.

A total of 10 lipid oxidation products were upregulated in the 50 °C and 60 °C groups at 16 d (Figure 5A1,A2). Among these, nonanal showed the greatest upregulation (Log_2_FC = 1.74), while tetradecane exhibited the highest VIP value (VIP = 1.75). The differential lipid oxidation products included decanal, 2,4-decadienal, hexadecenal, hexanal, ethyl decanoate, ethyl hexanoate, tetradecane, and hexadecane. In the 60 °C and 70 °C groups at 16 days (Figure 5B1,B2), seven lipid oxidation products were upregulated, with hexanal showing the greatest upregulation (Log_2_FC = 2.05) and the highest VIP value (VIP = 2.32). Differentially expressed products included nonanal, 2,4-decadienal, hexadecenal, and hexanal. At 20 d, 10 lipid oxidation products were upregulated in the 50 °C and 60 °C groups (Figure 5C1,C2). Hexanal again showed the greatest upregulation (Log_2_FC = 2.21) and the highest VIP value (VIP = 2.11). Differential lipid oxidation products at this stage included tetradecane, hexanal, octanal, ethyl hexanoate, and ethyl decanoate. In the 60 °C and 70 °C groups at 20 d (Figure 5D1,D2), six lipid oxidation products were upregulated, with nonanal showing the highest upregulation (Log_2_FC = 2.94) and hexanal the highest VIP value (VIP = 2.36). Differentially expressed lipid oxidation products included nonanal, hexanal, ethyl hexanoate, ethyl decanoate, hexadecenal, 1-octen-3-ol, and octenal. At 24 d, six lipid oxidation products were upregulated in the 50 °C and 60 °C groups (Figure 5E1,E2). Among these, hexadecane showed the highest upregulation (Log_2_FC = 0.74), while 2,4-decadienal exhibited the highest VIP value (VIP = 1.84). Differential lipid oxidation products at this stage included 2,4-decadienal, octanal, nonanal, ethyl decanoate, hexanal, and hexadecenal. In the 60 °C and 70 °C groups at 24 d (Figure 5F1,F2), four lipid oxidation products were upregulated, with decanal showing the greatest upregulation (Log_2_FC = 2.07) and nonanal exhibiting the highest VIP value (VIP = 2.06). Differential lipid oxidation products included nonanal, octanal, hexanal, and 1-octen-3-ol. In summary, the key differential lipid-derived volatile compounds were aldehydes, including decanal, nonanal, (E,E)-2,4-decadienal, hexanal, and (E)-2-hexadecenal; esters, such as ethyl decanoate and ethyl hexanoate; alkanes, namely tetradecane and hexadecane; and the alcohol 1-octen-3-ol. These compounds were primarily responsible for the characteristic flavor differences observed among the treatments.

### 3.4. Correlation Analysis Between Differential Lipid Oxidation Products and Histamine Content

**Figure 6:**
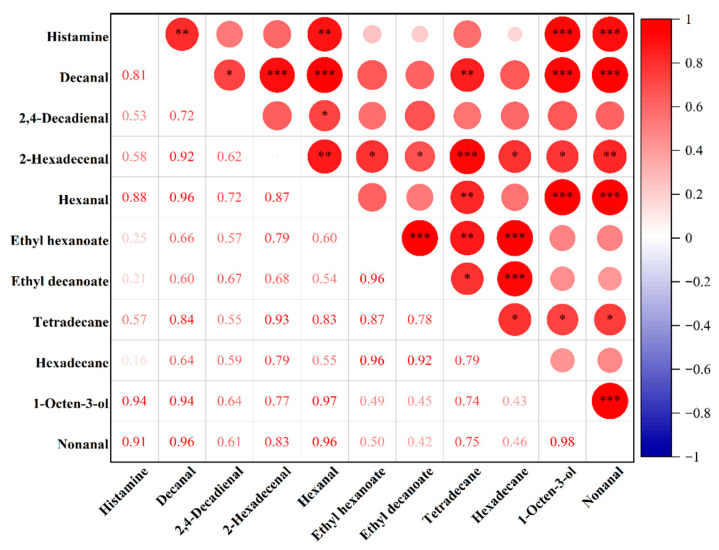
Heatmap of the correlation between lipid oxidation products and histamine content. Notes: ***, **, and * represent *p* ≤ 0.0001, *p* ≤ 0.01, and *p* ≤ 0.05, respectively.

To explore the relationship between different lipid oxidation products and histamine content, correlation analysis was performed between the differentially expressed lipid oxidation products identified through PCA and OPLS-DA and the measured histamine levels. As shown in Figure 6, four lipid oxidation products exhibited significant positive correlations with histamine content: decanal, nonanal, hexanal, and 1-octen-3-ol, with correlation coefficients of 0.81 (*p* < 0.01), 0.91 (*p* < 0.01), 0.88 (*p* < 0.01), and 0.94 (*p* < 0.01), respectively. These results suggest that these compounds may be closely associated with histamine formation in fermented sausages. Other products, such as 2,4-decadienal, also showed positive correlations, although the strength of the association was weaker (r = 0.53).

Accelerated lipid oxidation can trigger protein oxidation, which increases surface hydrophobicity and alters secondary structures. These changes further enhance protein degradation and supply precursor substrates for the formation of biogenic amines. [27,28]. Zamora et al. [4] demonstrated in pure culture systems that phenylalanine can undergo decarboxylation to form β-phenylethylamine in the presence of common lipid oxidation products found in foods, such as methyl 13-hydroperoxyoctadec-9,11-dienoate, 2,4-decadienal, 4,5-epoxy-2-decanal, 4-hydroxy-2-nonenal, and 4-oxo-2-nonenal. Similarly, Hidalgo et al. [5] reported that histidine can be decarboxylated to form histamine in the presence of lipid oxidation products. Among these, 2,4-alkadienals were identified as the most reactive toward histidine decarboxylation, exhibiting a lower activation energy requirement to promote histamine formation. The aldehydes identified in the present study do not fully align with those reported in the literature, which may be attributed to differences in the sources of lipid oxidation products. The lipid oxidation products examined in previous studies were specific compounds obtained through chemical synthesis or commercial sources, whereas those in this study were naturally derived from the oxidation of pork backfat. Moreover, correlation analysis revealed strong interrelationships among different lipid oxidation products, with several showing correlation coefficients exceeding 0.9. For example, the correlation coefficient between ethyl hexanoate and hexadecane reached 0.96, suggesting that increases in their concentrations during maturation may jointly contribute to elevated histamine content.

The formation of histamine in fermented meat products primarily results from the decarboxylation of histidine catalyzed by histidine decarboxylase secreted by amine-producing microorganisms, with histidine decarboxylase (hdcA) and histidine/histamine antiporter (hdcP) being the key regulatory genes—a mechanism that is currently well established [29,30]. Furthermore, the impact of lipid oxidation and its derived products on histamine accumulation in fermented meat products should not be overlooked. To date, the specific effects of lipid oxidation products on histamine formation, along with the underlying mechanisms, remain insufficiently elucidated. The present study demonstrates a clear correlation between the degree of lipid oxidation and histamine content, and further identifies several lipid oxidation products that may promote histamine formation in fermented sausages. These findings provide novel insights and distinctive contributions to this field of research. In fact, there may be two potential pathways through which lipid oxidation products promote the formation of biogenic amines: On one hand, aldehydes (such as nonanal, decanal, hexanal, and other lipid oxidation products) can directly induce the decarboxylation of amino acids through a series of chemical reactions, leading to the formation of biogenic amines. The specific process involves the condensation of the carbonyl group of the aldehyde with the α-amino group of the amino acid, forming a Schiff base (imine). The electron-withdrawing effect of the imine bond weakens the chemical bond between the α-carbon of the amino acid and its carboxyl group, thereby promoting decarboxylation and ultimately resulting in the formation of the corresponding biogenic amines. On the other hand, aldehydes may act as stress factors, further stimulating the biogenic amine production activity of amine-producing microorganisms [31,32]. Studies have shown that certain aldehyde compounds (such as 4-hydroxy-2-nonenal, 4-HNE) can act as signaling molecules to promote the expression of specific genes or regulate transcription factor activity by forming adducts with proteins, thus influencing gene expression. Therefore, this study hypothesizes that lipid oxidation products such as nonanal may also function as signaling molecules, potentially upregulating the expression of the histidine decarboxylase gene and its protein in amine-producing microorganisms, thereby promoting histamine formation [33,34].

Taken together, these findings indicate that both the interactions among lipid oxidation products and their correlations with histamine content emphasize the critical role of lipid oxidation processes in promoting histamine accumulation during fermented sausage maturation. Although the degree of oxidation selected in this study was relatively extreme, the final results indicate that lipid oxidation is positively correlated with histamine content to a certain extent. Therefore, in the food industry, it is necessary to control excessive lipid oxidation through measures such as temperature control, inoculation with starter cultures, and the addition of antioxidants.

## 4. Conclusions

This study examined the impact of lipid oxidation levels on histamine formation in fermented sausages and identified specific lipid oxidation products that promote histamine accumulation. The findings revealed a significant positive correlation between the extent of lipid oxidation and histamine content in fermented sausages (*p* < 0.05). In total, 33 lipid oxidation products were detected. Through multivariate statistical analyses, including principal component analysis (PCA) and orthogonal partial least squares discriminant analysis (OPLS-DA), coupled with Pearson correlation analysis, four lipid oxidation products, including decanal, nonanal, hexanal, and 1-octen-3-ol, were determined to exhibit significant positive correlations with histamine content (*p* < 0.05). These compounds were pinpointed as the primary contributors to histamine formation in fermented sausages.

Overall, this research substantiates the facilitative role of lipid oxidation products in histamine generation within fermented sausage systems. It offers a theoretical basis and practical insights for mitigating biogenic amine accumulation by modulating lipid oxidation processes, thereby enhancing the safety and quality of fermented meat products. These results hold substantial implications for refining fermentation protocols and bolstering food safety measures in fermented meats. Future investigations should delve deeper into the precise mechanisms and regulatory pathways of individual lipid oxidation products to enable more targeted control of biogenic amine formation.

## Figures and Tables

**Table 1:** Percentage of Main Lipid Oxides in Fermented Sausages.

Type	Name	16 d			20 d			24 d		
		50 °C	60 °C	70 °C	50 °C	60 °C	70 °C	50 °C	60 °C	70 °C
Aldehydes	Nonanal	0.43 ± 0.02 ^d^	1.47 ± 0.14 ^c^	3.49 ± 0.12 ^a^	0.14 ± 0.05 ^d^	0.46 ± 0.07 ^d^	3.46 ± 0.62 ^ab^	0.7 ± 0.08 ^d^	1.15 ± 0.32 ^c^	3.65 ± 0.23 ^ab^
	Decanal	1.42 ± 0.11 ^f^	3.12 ± 0.07 ^cd^	4.82 ± 1.04 ^a^	2.42 ± 0.18 ^e^	4.82 ± 0.48 ^ab^	4.92 ± 0.92 ^a^	0.85 ± 0.02 ^g^	1.28 ± 0.08 ^fg^	3.82 ± 0.12 ^cd^
	2,4-decadienal	0.55 ± 0.01 ^e^	1.25 ± 0.01 ^cd^	2.75 ± 0.13 ^a^	1.55 ± 0.18 ^c^	2.75 ± 0.33 ^ab^	1.85 ± 0.04 ^b^	0.42 ± 0.03 ^e^	1.75 ± 0.11 ^bc^	2.25 ± 0.13 ^a^
	2-Hexadecenal	0.48 ± 0.03 ^d^	1.18 ± 0.01 ^c^	2.88 ± 0.62 ^a^	0.48 ± 0.02 ^d^	0.88 ± 0.01 ^cd^	1.98 ± 0.12 ^b^	0.38 ± 0.01 ^d^	0.68 ± 0.07 ^cd^	1.18 ± 0.01 ^c^
	Hexanal	0.45 ± 0.01 ^e^	1.15 ± 0.12 ^d^	4.85 ± 0.07 ^a^	0.65 ± 0.01 ^e^	2.85 ± 0.22 ^c^	4.95 ± 0.59 ^a^	0.68 ± 0.02 ^e^	1.08 ± 0.06 ^de^	2.58 ± 0.17 ^bc^
Ketones	Acetylacetone	1.49 ± 0.04 ^b^	1.19 ± 0.01 ^c^	1.79 ± 0.06 ^a^	1.04 ± 0.04 ^cd^	1.55 ± 0.01 ^b^	1.21 ± 0.05 ^c^	0.43 ± 0.01 ^g^	0.78 ± 0.03 ^ef^	1.22 ± 0.08 ^c^
	Caryophyllene	1.61 ± 0.1 ^a^	1.21 ± 0.06 ^c^	1.33 ± 0.04 ^bc^	1.14 ± 0.05 ^cd^	1.55 ± 0.01 ^ab^	1.24 ± 0.03 ^c^	0.58 ± 0.03 ^e^	0.95 ± 0.01 ^d^	1.45 ± 0.07 ^bc^
	Heptadecanone	1.57 ± 0.13 ^ab^	1.27 ± 0.06 ^cde^	1.67 ± 0.02 ^a^	1.57 ± 0.02 ^ab^	1.67 ± 0.01 ^ab^	1.77 ± 0.06 ^a^	0.52 ± 0.04 ^g^	0.88 ± 0.01 ^f^	1.37 ± 0.03 ^bcde^
Esters	Ethylhexanoate	1.51 ± 0.16 ^g^	3.21 ± 0.53 ^de^	4.71 ± 0.09 ^ab^	2.51 ± 0.12 ^f^	3.44 ± 0.43 ^cd^	4.81 ± 0.17 ^a^	0.44 ± 0.01 ^h^	0.77 ± 0.08 ^h^	1.2 ± 0.01 ^g^
	Ethyldecanoate	1.63 ± 0.01 ^g^	3.33 ± 0.12 ^de^	4.63 ± 0.08 ^ab^	2.63 ± 0.05 ^f^	3.54 ± 0.18 ^cd^	4.73 ± 0.11 ^ab^	0.62 ± 0.01 ^h^	1.02 ± 0.02 ^h^	1.52 ± 0.01 ^g^
Alcohols	1-Hexanol	0.00 ± 0.00 ^g^	0.96 ± 0.02 ^d^	1.38 ± 0.21 ^c^	0.00 ± 0.00 ^g^	0.21 ± 0.48 ^f^	1.48 ± 0.42 ^b^	0.00 ± 0.00 ^g^	0.46 ± 0.02 ^e^	1.65 ± 0.14 ^a^
	1-Octen-3-ol	0.00 ± 0.00 ^g^	0.23 ± 0.01 ^d^	0.89 ± 0.06 ^b^	0.00 ± 0.00 ^g^	0.16 ± 0.04 ^e^	0.92 ± 0.03 ^a^	0.00 ± 0.00 ^g^	0.29 ± 0.11 ^c^	0.96 ± 0.18 ^a^
Alkanes	Tetradecane	1.39 ± 0.14 ^e^	3.09 ± 0.21 ^c^	4.82 ± 0.31 ^a^	2.39 ± 0.19 ^d^	4.82 ± 0.51 ^ab^	4.92 ± 0.65 ^a^	0.22 ± 0.01 ^f^	0.46 ± 0.01 ^f^	0.79 ± 0.06 ^f^
	Hexadecane	1.72 ± 0.08 ^f^	3.42 ± 0.91 ^d^	4.48 ± 1.02 ^ab^	2.72 ± 0.62 ^e^	3.01 ± 0.17 ^de^	3.58 ± 0.46 ^cd^	0.76 ± 0.05 ^g^	1.15 ± 0.03 ^g^	1.65 ± 0.08 ^fg^

Notes: Different lowercase letters indicate significant differences (*p* < 0.05) among distinct groups in the same fermentation time.

## Data Availability

The original contributions presented in this study are included in the article/Supplementary Materials. Further inquiries can be directed to the corresponding author.

## Supplementary Materials

The following supporting information can be downloaded at https://www.mdpi.com/article/10.3390/foods14234166/s1, Table S1: Percentage of minor lipid oxides in fermented sausages.
