# Supplementary material for: Effect of Lipid Oxidation Products on Histamine Formation in Fermented Sausages
Source: Foods. 2025 Dec 4;14(23):4166. doi: 10.3390/foods14234166 (PMC12691741; doi:10.3390/foods14234166)
Supplement: Supplementary file 1 [file foods-14-04166-s001.zip › foods-3977840-supplementary.pdf]

Table S1 Percentage of Minor Lipid Oxides in Fermented Sausages.

Type	Name	16 d			20 d			24 d		
		50° C	60° C	70° C	50° C	60° C	70° C	50° C	60° C	70° C
Aldehydes	Hexadecanal	1.62 ± 0.02^{ab}	1.32 ± 0.01^{bc}	1.62 ± 0.01^{ab}	1.62 ± 0.37^{ab}	1.62 ± 0.22^{ab}	1.2 ± 0.12^{cd}	0.72 ± 0.07^{e}	1.15 ± 0.31^{cd}	1.62 ± 0.01^{ab}
	Eicosanal	1.58 ± 0.05^{cd}	1.28 ± 0.01^{d}	1.68 ± 0.01^{a}	1.58 ± 0.11^{e}	1.68 ± 0.02^{bc}	1.78 ± 0.01^{ab}	0.45 ± 0.07^{a}	0.82 ± 0.03^{e}	1.28 ± 0.22^{f}
Ketones	Thioxanthene	0.52 ± 0.01^{cd}	0.22 ± 0.02^{fg}	0.72 ± 0.01^{bc}	0.52 ± 0.04^{cd}	0.72 ± 0.04^{bc}	0.82 ± 0.01^{b}	0.39 ± 0.06^{efg}	0.72 ± 0.01^{bc}	1.12 ± 0.11^{a}
	Cycloheptadienone	0.04 ± 0.01^{e}	0.17 ± 0.01^{bc}	0.24 ± 0.02^{ab}	0.09 ± 0.01^{de}	0.13 ± 0.01^{cd}	0.15 ± 0.01^{cd}	0.01 ± 0.00^{e}	0.17 ± 0.02^{bc}	0.08 ± 0.01^{de}
	2,3-Octanedione	0.01 ± 0.00^{d}	0.04 ± 0.00^{cd}	0.14 ± 0.01^{ab}	0.04 ± 0.00^{cd}	0.07 ± 0.00^{bcd}	0.14 ± 0.01^{abc}	0.03 ± 0.00^{cd}	0.07 ± 0.01^{bcd}	0.35 ± 0.08^{a}
Esters	Ethyldodecanoate	1.14 ± 0.02^{ef}	1.54 ± 0.06^{cd}	1.66 ± 0.08^{bc}	1.74 ± 0.12^{ab}	1.77 ± 0.01^{ab}	1.72 ± 0.05^{ab}	0.37 ± 0.01^{h}	0.65 ± 0.05^{g}	1.05 ± 0.01^{f}
	Ethyltetradecanoate	0.31 ± 0.02^{fg}	0.35 ± 0.01^{efg}	0.45 ± 0.01^{cde}	0.41 ± 0.01^{def}	0.48 ± 0.05^{cde}	0.44 ± 0.01^{def}	0.32 ± 0.01^{efg}	0.58 ± 0.01^{bcd}	0.92 ± 0.03^{a}
	Ethylhexadecanoate	0.44 ± 0.07^{def}	0.54 ± 0.01^{cde}	0.74 ± 0.06^{bc}	0.47 ± 0.01^{ef}	0.49 ± 0.07^{def}	0.37 ± 0.01^{fg}	0.38 ± 0.01^{fg}	0.67 ± 0.01^{bcd}	1.08 ± 0.06^{a}
	Ethylhexadecenoate	0.24 ± 0.02^{fg}	0.54 ± 0.06^{de}	0.67 ± 0.07^{cd}	0.73 ± 0.08^{bc}	0.76 ± 0.01^{b}	0.64 ± 0.01^{cd}	0.35 ± 0.01^{efg}	0.62 ± 0.05^{cd}	1.02 ± 0.02^{a}
	Linalylacetate	0.45 ± 0.03^{ef}	0.24 ± 0.01^{g}	0.31 ± 0.01^{fg}	0.78 ± 0.07^{cd}	0.4 ± 0.01^{ef}	0.98 ± 0.08^{bc}	0.56 ± 0.05^{de}	0.92 ± 0.11^{c}	1.42 ± 0.06^{a}
	Ethylp-methoxycinnamate	0.84 ± 0.02^{d}	0.99 ± 0.03^{cd}	1.21 ± 0.08^{bc}	0.49 ± 0.01^{e}	0.89 ± 0.05^{d}	2.01 ± 0.16^{a}	0.4 ± 0.01^{e}	0.7 ± 0.05^{d}	1.1 ± 0.07^{c}
	Diethylphthalate	0.44 ± 0.02^{g}	0.47 ± 0.05^{g}	0.58 ± 0.01^{fg}	0.46 ± 0.03^{de}	0.46 ± 0.01^{g}	0.87 ± 0.06^{g}	0.67 ± 0.04^{fg}	1.08 ± 0.01^{cd}	1.58 ± 0.06^{ab}
**Acid**	Aceticacid	0.00 ± 0.00^{a}	0.00 ± 0.00^{a}	0.01 ± 0.00^{a}	0.00 ± 0.00^{a}	0.00 ± 0.00^{a}	0.06 ± 0.00^{a}	0.00 ± 0.00^{a}	0.00 ± 0.00^{a}	0.01 ± 0.00^{a}
	Hexanoicacid	0.00 ± 0.00^{a}	0.00 ± 0.00^{a}	0.00 ± 0.00^{a}	0.00 ± 0.00^{a}	0.00 ± 0.00^{a}	0.01 ± 0.00^{a}	0.00 ± 0.00^{a}	0.00 ± 0.00^{a}	0.01 ± 0.00^{a}
Alcohols	1-Octanol	0.00 ± 0.00^{g}	0.76 ± 0.02^{f}	1.21 ± 0.06^{c}	0.00 ± 0.00^{g}	0.93 ± 0.02^{e}	1.87 ± 0.08^{b}	0.00 ± 0.00^{g}	0.96 ± 0.08^{d}	2.01 ± 0.38^{a}
	Cycloheptanol	0.00 ± 0.00^{f}	0.71 ± 0.05^{e}	0.86 ± 0.07^{c}	0.00 ± 0.00^{f}	0.81 ± 0.01^{c}	0.89 ± 0.05^{ab}	0.00 ± 0.00^{f}	0.92 ± 0.02^{a}	0.86 ± 0.03^{bc}
Alkanes	Eicosane	1.38 ± 0.05^{c}	3.08 ± 0.71^{a}	3.14 ± 0.12^{a}	0.46 ± 0.02^{d}	1.31 ± 0.07^{c}	1.92 ± 0.04^{b}	0.22 ± 0.01^{d}	0.46 ± 0.01^{d}	0.79 ± 0.01^{d}
	Heptadecane	0.12 ± 0.02^{d}	0.6 ± 0.09^{b}	0.89 ± 0.01^{a}	0.09 ± 0.01^{d}	0.31 ± 0.01^{cd}	0.33 ± 0.01^{cd}	0.14 ± 0.01^{d}	0.4 ± 0.01^{bcd}	0.66 ± 0.04^{b}
	Eicosene	0.48 ± 0.01^{b}	0.4 ± 0.09^{b}	0.1 ± 0.01^{cd}	0.34 ± 0.07^{bc}	0.77 ± 0.02^{a}	0.45 ± 0.01^{b}	0.2 ± 0.01^{cd}	0.23 ± 0.03^{cd}	0.21 ± 0.02^{cd}

Notes: Different lowercase letters indicate significant differences ($P < 0.05$) among distinct groups for the same fermentation time.
